# On Time Domain Analysis of Photoplethysmogram Signals for Monitoring Heat Stress

**DOI:** 10.3390/s151024716

**Published:** 2015-09-25

**Authors:** Mohamed Elgendi, Rich Fletcher, Ian Norton, Matt Brearley, Derek Abbott, Nigel H. Lovell, Dale Schuurmans

**Affiliations:** 1 Electrical and Computer Engineering in Medicine Group, University of British Columbia and BC Children’s Hospital, Vancouver, BC V6H 3N1, Canada; 2 Department of Computing Science, University of Alberta, Edmonton, AB T6G 2E8, Canada; E-Mail: daes@ualberta.ca; 3 Media Lab, Massachusetts Institute of Technology, Boston, MA 02139, USA; E-Mail: fletcher@media.mit.edu; 4 National Critical Care and Trauma Response Centre, Darwin, NT 0810, Australia; E-Mails: nortoni@who.int (I.N.); matt.brearley@nt.gov.au (M.B.); 5 School of Electrical and Electronic Engineering, University of Adelaide, Adelaide, SA 5005, Australia; E-Mail: derek.abbott@adelaide.edu.au; 6 Graduate School of Biomedical Engineering, UNSW, Sydney, NSW 2052, Australia; E-Mail: n.lovell@unsw.edu.au

**Keywords:** global warming, affordable healthcare, thermal stress

## Abstract

There are a limited number of studies on heat stress dynamics during exercise using the photoplethysmogram (PPG) and its second derivative (APG). However, we investigate the most suitable index from short PPG signal recordings for heat stress assessment. The APG waveform consists of *a*, *b*, *c* and *d* waves in systole and an *e* wave in diastole. Our preliminary results indicate that the use of the energy of aa area, derived from PPG signals measured from emergency responders in tropical conditions, is promising in determining the heat stress level using 20-s recordings. After examining 14 time domain features using leave-one-out cross-validation, we found that the aa energy extracted from PPG signals is the most informative feature for classifying heat-stressed subjects, with an overall accuracy of 79%. Moreover, the combination of the aa energy with the traditional heart rate variability index of heat stress (*i.e.*, the square root of the mean of the squares of the successive aa intervals) improved the heat stress detection to an overall accuracy of 83%.

## 1. Introduction and Motivation

### 1.1. Heat Stress

According to the Intergovernmental Panel on Climatic Change (IPCC), accelerated global warming is predicted due to increasing anthropogenic greenhouse gas emissions. Interestingly, global warming is anticipated to have a long-term impact on human health. More specifically, there is a 3% increase in death rates per 1 °C increase in temperature, for all causes of mortality, in hot and arid regions where the temperature of the warmest months exceeds 30 °C [1]. This increase in mortality can be attributed to the exposure to excessive heat that is straining on the body’s cooling system, known as heat stress. During an episode of heat stress, the body gains heat faster than it can dissipate it. Heat stress can lead to heat-related illness, disability and even death when combined with other factors, such as hard physical activity, fatigue and some medical conditions.

The literature identifies two main heat stress indices: body core temperature (BCT) and heart rate variability (HRV). In terms of BCT, in 2002, the National Athletic Trainers’ Association recommended measuring the rectal temperature as a criterion standard for recognizing exertional heat stroke [2]. Five years later, Casa *et al.* [3] found that the gastrointestinal temperature was the only measurement that accurately assessed BCT, rather than rectal temperature (the criterion standard). Moreover, they found out that the oral, axillary, aural, temporal and field forehead temperatures were significantly different from the rectal temperature and, therefore, are considered invalid for assessing hyperthermia in individuals exercising outdoors in the heat.

Regarding HRV, Bruce-Low *et al.* [4] reported that when subjects are continuously subjected to dry heat, the heat induces a stress response as indicated by a significantly increased heart rate (HR). This increase in HR appears to occur through a significant reduction in parasympathetic control of HR indicated by reduced RMSSD (the root mean square of the differences of successive differences).

Using these two heat stress indices is not practical, as measuring the BCT has an invasive nature, and calculating HRV requires long recorded electrocardiogram signals. Therefore, there is a need to develop an easier and non-invasive method to assess heat stress to prevent the onset of adverse outcomes related to heat stress and heat-related deaths.

### 1.2. PPG Analysis

Photoplethysmogram (PPG) signal collection is a simple-to-measure and non-invasive method that can be carried out using a fingertip. The PPG collected from the fingertip can potentially be utilized to detect heat stress, leading to the creation of a new measurement methodology for heat stress. It is worth noting that the sympathetic activation caused by stress can also be measured using skin conductance [5]. However, this is not a feasible option for heat stress detection due to variations in skin sweating. Therefore, PPG measurement that can be measured in an ambulatory setting represents an attractive option. As Rowell *et al.* [6] found reductions in cardiac output, central blood volume and stroke volume during heat stress, we expect that the morphology of the PPG waveform will change accordingly. Recent improvements in wearable sensor technology allow for the continuous measurement of PPG for the purpose of measuring heart rate. However, information derived from the PPG signal can go beyond exclusive heart rate measurement to provide additional information on physiological and autonomic arousal and stress conditions.

Our recent investigation to detect systolic peaks in PPG signals measured after exercise showed some challenges due to motion artifacts, sweat and non-stationary effects [7]. Several filters and algorithms have been examined to analyze of the PPG wave contour; however, they still lack accuracy and reproducibility [8]. As a result of these challenges, researchers have started to apply the second derivative to emphasize and easily quantify the delicate changes in the PPG contour [9]. For these reasons, a second derivative will be used in this project to increase accuracy.

**Figure 1:**
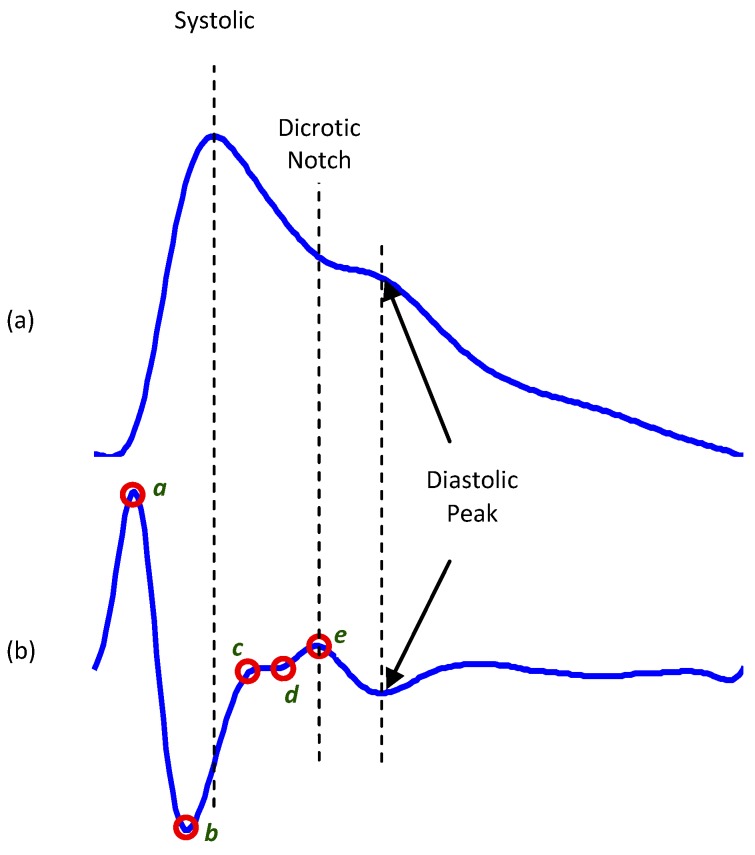
Fingertip photoplethysmogram signal morphology [12]. (**a**) Fingertip photoplethysmogram; (**b**) second derivative wave of photoplethysmogram. The photoplethysmogram waveform consists of one systolic wave and one diastolic wave, while the second derivative photoplethysmogram waveform consists of four systolic waves (*a*, *b*, *c*, and *d* waves) and one diastolic wave (*e* wave).

The acronym for the second derivative of the PPG is usually SDPPG or APG; however, APG will be used exclusively in this study, according to the recommendations in [10]. As shown in Figure 1b, the APG waveform consists of four systolic waves (*a*, *b*, *c*, and *d* waves) and one diastolic wave (*e* wave) [11]. The height of each wave was measured from the baseline, with the values above the baseline being positive and those under it negative.

The relative heights of these waves (b/a, c/a, d/a and e/a ratios), particularly the b/a ratio, are related to aging and carotid distensibility [13] and are used in calculating the aging index (b‒c‒d‒e)/a [14]. Recently, the detection of *a* waves in APG signals has been used to calculate heart rate [15,16] and heart rate variability indices [17,18,19]. Despite the application of APG to cardiac variables and that the clinical significance of APG measurement has been well investigated, there is still a lack of studies focusing on heat stress assessment using APG signals. Matsuyama [20] assessed the most suitable index for heat stress assessment in APG signals, determining that the typical indicators of APG analysis, such as c/a, d/a and e/a, are insufficient for heat stress assessment, as they are very difficult to detect correctly: *c*, *d* and *e* waves merge after heat stress [21]. Therefore, our investigation is focused on examining time domain features based on systolic references in heat-stressed PPG and APG signals, especially after the automatic detection of *a* and *b* waves became feasible [22].

## 2. Materials and Methods

### 2.1. Data Collection

**Figure 2:**
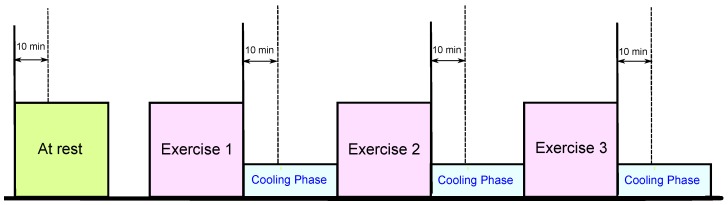
Measurement protocol. The duration of the whole experiment was approximately 4 h; each exercise consumed approximately 30 min, while the photoplethysmogram (PPG) signals were collected during the 30-min break of each exercise.

The heat stress PPG data for this study were collected as part of a National Critical Care and Trauma Response Centre (NCCTRC) project to assess the physiological and perceptual responses of emergency responders to a simulated chemical, biological and radiological (CBR) incident in tropical environmental conditions, to compare the efficacy of various cooling methods. The background of the NCCTRC’s thermal research can be found in [23]. Forty healthy, heat-acclimatised emergency responders (30 males and 10 females) with a mean ± SD age of 34.7 ± 6.6 volunteered and provided written informed consents to participate in this study, which was approved by the Human Research Ethics Committee of the Northern Territory Department of Health and Menzies School of Health Research. Participants undertook 30 min of triaging and resuscitating, transporting and decontaminating weighted manikins, while wearing Level 3 personal protective equipment, which comprised a fully-enclosed, impermeable suit, including, boots, gloves, hood, face mask and respirator (SE400i, S.E.A. Group, Warriewood, Australia), followed by 30 min of rest and cooling. This protocol was repeated three times with PPG data collected during each rest period, as shown in Figure 2. The database is available upon request at Charles Darwin University: http://www.cdu.edu.au/ehse.

Here, PPG data were measured by a photoplethysmography-equipped device (Salus APG, Osaka, Japan) at a sampling rate of 367 Hz, with the sensor located at the cuticle of the second digit of the left hand. Measurements were taken for 20 s while participants were undertaking seated rest. An emergency physician annotated the systolic peaks as controls for evaluation. The participants were normotensive (mean systolic blood of 129.3 mmHg, range 110–165 mmHg) and had no known cardiovascular, neurological or respiratory disease. Prior to the experiment, the participants provided information about their physical condition. Physical information, such as height and weight, were also measured for reference and summarized in Table 1. Alcohol consumption and smoking were eliminated during 24 h and 2 h before experimentation, respectively. For signal analysis, MATLAB 2010b (The MathWorks, Inc., Natick, MA, USA) was used. An Omron HEM-907 was used for blood pressure measurement.

**Table 1:** Demographic data for the participants in this study.

Characteristic	Mean	Standard Deviation
Age (years)	34.7	6.6
Body Mass (kg)	81.8	12.8
Height (cm)	176.0	6.5
Body Mass Index ($\text{kg}\cdot m^{‒2}$)	26.3	3.6
Resting Systolic Blood Pressure (mmHg)	129.3	13.3
Resting Heart Rate (bpm)	76.0	14.7
Resting Core Temperature (°C)	37.4	0.4
After Exercise 1 Systolic Blood Pressure (mmHg)	144.4	22.4
After Exercise 1 Heart Rate (bpm)	133.1	29.9
After Exercise 1 Core Temperature (°C)	38.4	0.5
After Exercise 2 Systolic Blood Pressure (mmHg)	144.6	20.6
After Exercise 2 Heart Rate (bpm)	143.9	23.6
After Exercise 2 Core Temperature (°C)	38.0	0.9
After Exercise 3 Systolic Blood Pressure (mmHg)	132.7	13.5
After Exercise 3 Heart Rate (bpm)	138.2	25.7
After Exercise 3 Core Temperature (°C)	37.8	0.8

### 2.2. Methodology

In this study, our goal is to find out the most informative feature that can be used for heat stress assessment. Furthermore, we investigate whether the PPG or APG waveform potentially improves the heat stress analysis.

#### 2.2.1. APG Signal

**Figure 3:**
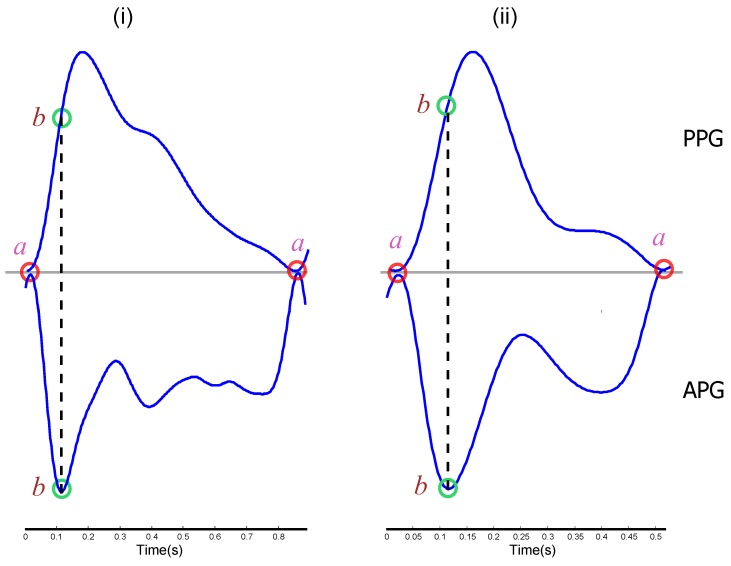
Projection of *a* and *b* waves on the aa area of the fingertip photoplethysmogram signal (PPG) and its second derivative (APG). (**i**) One heart beat of the PPG and its APG measured from a subject at rest; (**ii**) one heart beat of the PPG and its APG measured from a subject after simulated heat stress induction.

To produce an informative APG signal, two steps are required: filtering the PPG and differentiating the filtered PPG. We applied a zero-phase second-order Butterworth filter, with a 0.5–7 Hz bandpass based on the optimization process in [22]. Then, the second derivative was applied to the filtered PPG to analyze the APG waveform. Equations (1) and (2) represent a non-causal filter; the three-point center derivative was created with a delay of only two samples, as follows:

(1) $$S^{\prime }\left[n\right]=\frac{dS}{dt}{|}_{t=nT}=\frac{1}{2T}\left(S\left[n+1\right]‒S\left[n‒1\right]\right)$$

(2) $$\;\;\;Z\left[n\right]=\frac{dS^{\prime }}{dt}{|}_{t=nT}=\frac{1}{2T}\left(S^{\prime }\left[n+1\right]‒S^{\prime }\left[n‒1\right]\right)$$

where *T* is the sampling interval and equals the reciprocal of the sampling frequency and *n* is the number of data points. Figure 3 shows the second derivative (bottom figures) of the filtered PPG signal (top figures) measured at rest and after exercise.

#### 2.2.2. Feature Extraction

To our knowledge, this is the first study to investigate the correlation of PPG features with the effect of heat stress. Therefore, there was no feature tested before to capture the heat stress impact on PPG signals. To start this line of research, it was logical to examine existing features used in the literature to diagnose different diseases, such as the b/a index, the amplitude of the *a* wave and the amplitude of the *b* wave. Furthermore, we tested new features to determine the optimal feature for heat stress detection, such as the energy of the aa area, the energy of the ab area, the energy of the ba area and the slope of the ab segment. In total, we tested 14 time domain features, seven features extracted from the PPG signals and seven features extracted from the APG signals, which can be defined in a compact manner as follows:

(3) $$f_{1,S}=\sum_{n=1}^{N}S{\left(A\left[n\right]:B\left[n\right]\right)}^{2}$$

(4) $$f_{2,S}=\sum_{n=1}^{N}S{\left(A\left[n\right]:A\left[n+1\right]\right)}^{2}$$

(5) $$f_{3,S}=\sum_{n=1}^{N}S{\left(B\left[n\right]:A\left[n+1\right]\right)}^{2}$$

(6) $$f_{4,S}=S\left(A\left[n\right]\right)$$

(7) $$f_{5,S}=S\left(B\left[n\right]\right)$$

(8) $$f_{6,S}=S\left(B\left[n\right]\right)/S\left(A\left[n\right]\right)$$

(9) $$f_{7,S}=\left(S\left(B\left[n\right]\right)‒S\left(A\left[n\right]\right)\right)/\left(B\left[n\right]‒A\left[n\right]\right)$$

where *S* refers to the either PPG or APG signal, *N* refers to the total number of beats in the processed signal and *A* and *B* arrays contain the annotated *a* waves and *b* waves, respectively, in each APG signal. Colon notation is used to specify sequences, for example S(x:y) is a vector of PPG or APG data points from *x* to *y*.

As the heart rate may significantly increases due to heat stress, we calculated beat-to-beat intervals from the APG signals using *a* waves [16]. The annotated *a* waves are used to calculate the duration of each consecutive aa interval as follows:

(10) aa[n]=A[n+1]‒A[n]

where *A* contains the annotated *a* waves in each APG signal, *n* is the number of *a* waves and aa contains the *a*–*a* intervals.

#### 2.2.3. Statistical Analysis

We calculated seven features from each PPG and APG signal recording. As we had 40 subjects, each feature set contains 80 values. Each feature set consisted of 40 values calculated from subjects measured before the simulated heat stress induction and 40 values calculated from subjects measured after the simulated heat stress induction. As we have a small sample size, we compared the values within each feature set by applying the paired Mann–Whitney test (p<0.05 was considered significant). Because we considered all of these features simultaneously, it is likely that a few *p*-values are small merely due to stochastic fluctuations rather than due to systematic differences between subjects measured before and after the simulated heat stress induction. As a consequence, the *p* values need to be appropriately corrected. One may try to control the probability that a false positive occurs by applying a Bonferroni post-correction [24]. As we are dealing with many different simultaneous tests (42 tests in total), it is more natural to try to control the false discovery (false positive) rate. Therefore, we used the Holm–Bonferroni method because it controls the false positive rate and is a simple test that is uniformly more powerful than the Bonferroni correction [25].

As the main objective of this research is to find the optimal time domain feature for assessing simulated heat stress induction, we tested four classifiers: Mahalanobis distance, linear discriminant analysis (LDA), quadratic discriminant analysis (QDA) and the linear support vector machine (SVM). The classification rates were calculated using leave-one-out cross-validation. Two statistical measures were used for the output of each classifier: sensitivity (SE), which was calculated using the formula TP/(TP+FN), and positive predictivity (PP), which was calculated using the formula TP/(TP+FN); whereas TP is the number of true positives (subjects measured after simulated heat stress induction detected in heat-stressed subjects); FN is the number of false negatives (subjects measured after simulated heat stress induction have not been detected as heat-stressed subjects); and FP is the number of false positives (subjects measured before simulated heat stress induction detected as heat-stressed subjects). To compare the performance of the features given the small dataset in each classifier, we applied the $F_{1}$ score, as recommended in [26], which is defined as 2×(SE×PP)/(SE+PP). The overall accuracy (OA) is the average of $F_{1}$ scores for all classifiers.

## 3. Results and Discussion

An ingestible telemetric temperature sensor (CorTemp 100, HTI Technologies, Florida, MI, USA) was used to measure core body temperature by transmitting a signal proportional to the temperature of the gastrointestinal tract to a handheld receiver. Once located in the gastrointestinal tract, the thermosensitive pill is a valid index of core temperature when referenced to rectal or esophageal temperature [27,28].

Participants ingested a core temperature pill with fluid prior to breakfast that preceded the commencement of data collection by approximately 4 h. This time frame was adopted to allow the pill to empty from the stomach and enter the small intestine while minimizing the risk of the pill being emptied from the body prior to the completion of the study [27]. To control for the possibility of local cooling of the telemetry pill via ingestion of fluids and/or crushed ice, subjects were excluded from the study if gastrointestinal temperature was less than 35.5 °C and/or decreased by 2 °C in any 5-min period during the rest phase.

**Figure 4:**
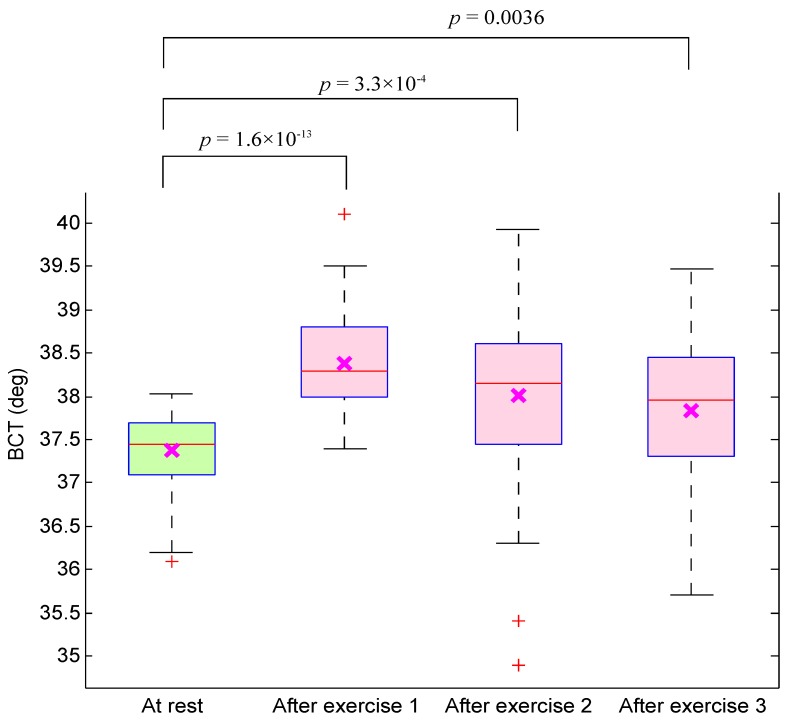
Boxplot of body core temperature (BCT) for subjects measured before and after exercise. The *p*-value was calculated using the paired Mann–Whitney test.

Figure 4 demonstrates a significant difference between at-rest measurement and those after exercises based on the BCT. We tried to collect the PPG signals immediately after exercise. However, some participants may have cooled down while queuing for measurement. Therefore, the obtained *p*-value from comparing at-rest measurement and the simulated heat stress simulations did not linearly decrease. For example, the returned *p*-value from the first BCT test (at rest *vs.* after exercise 1) was $1.6\times 10^{‒13}$, while the second test (at rest *vs.* after exercise 2) was $3.3\times 10^{‒4}$ and for the last test (at rest *vs.* after exercise 3) was 0.0036. Note, this step was carried out to ensure heat stress impact.

**Figure 5:**
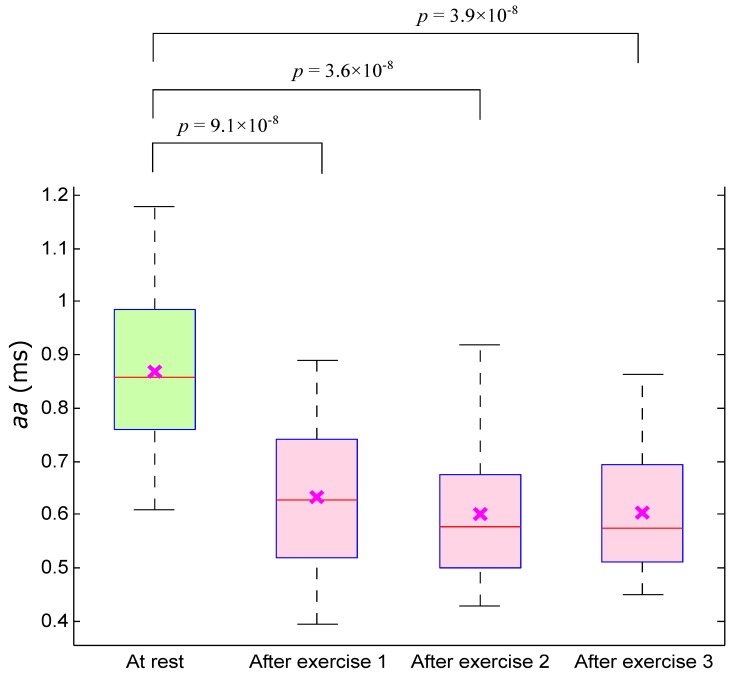
Boxplot of the aa interval for subjects measured before and after exercise. The *p*-value was calculated using the paired Mann–Whitney test.

It is expected that the aa interval will be shorter as the heart rate increases during exercise. Figure 5 illustrates the significant difference between the four measurements based on the average aa intervals per participant using the paired Mann–Whitney test. It is clear that the aa interval shows an overall significant difference (between at-rest measurement and those after exercise) compared to the BCT. The returned *p*-value from the first aa test (at rest *vs.* after exercise 1) was $9.1\times 10^{‒8}$, while the second test (at rest *vs.* after exercise 2) was $3.6\times 10^{‒8}$ and for the last test (at rest *vs.* after exercise 3) was $3.9\times 10^{‒8}$. However, the aa interval will be used in calculating the RMSSD. Therefore, we excluded the aa interval as a time domain feature from our investigation.

The separability performance of the discussed 14 time domain features for predicting heat stress is shown in Table 2. It is clear that the energy of the ba area of the PPG signal provides the lowest overall *p*-value ($4.1\times 10^{‒9}$), while the energy of the aa is the second lowest overall *p*-value ($4.8\times 10^{‒9}$). The lowest and second lowest features are very close in terms of separability. As the main goal of this study is to find the optimal feature with significant separability power, we examined the classification performance of each feature using four different classifiers, Mahalanobis, LDA, QDA and SVM, as shown in Table 3.

**Table 2:** Feature comparison in differentiating between before and after exercise measurements. Mean and standard deviation values of 14 features extracted from the PPG and APG waveforms. The *p*-value of discriminating between before exercise (BE) and after three exercises (E1, E2 and E3) is calculated using the Mann–Whitney test. The average of the three *p*-values ($\bar{p}$) is given in the last column. Uncorrected *p*-values from the Mann–Whitney test, where * and ** indicate p<0.05 and p<0.005, respectively; ${}^{†}$ indicates *p* values that remain significant with a p<0.05 after post-correction (Bonferroni–Holm, α<0.05).

Feature	Before Exercise (BE)	After Exercise (E1)	After Exercise (E2)	After Exercise (E3)	*p*-value	*p*-value	*p*-value	$\bar{p}$
					(BE *vs.* E1)	(BE *vs.* E2)	(BE *vs.* E3)	
$f_{1,\text{PPG}}$ (Energy ab)	95.516 ± 9.998	96.302 ± 10.000	101.245 ± 8.792	89.148 ± 8.822	0.648	$0.010^{*,†}$	$0.0047^{**,†}$	0.221
$f_{2,\text{PPG}}$ (Energy aa)	7.9$\times 10^{2}$ ± 1.3$\times 10^{2}$	5.8$\times 10^{2}$ ± 1.2$\times 10^{2}$	5.6$\times 10^{2}$ ± 1.2$\times 10^{2}$	5.6$\times 10^{2}$ ± 91	1.4$\times 10^{‒8**,†}$	4.4$\times 10^{‒10**,†}$	3.7$\times 10^{‒11**,†}$	4.8$\times 10^{‒9}$
$f_{3,\text{PPG}}$ (Energy ba)	7.0$\times 10^{2}$ ± 1.3$\times 10^{2}$	4.9$\times 10^{2}$ ± 1.2$\times 10^{2}$	4.6$\times 10^{2}$ ± 1.2$\times 10^{2}$	4.7$\times 10^{2}$ ± 93	1.2$\times 10^{‒8**,†}$	3.0$\times 10^{‒10**,†}$	1.3$\times 10^{‒10**,†}$	4.1$\times 10^{‒9}$
$f_{4,\text{PPG}}$ (Amp*b*)	−1.259 ± 0.222	−1.216 ± 0.198	−1.115 ± 0.260	−1.194 ± 0.202	0.264	$0.011^{*†}$	0.140	0.138
$f_{5,\text{PPG}}$ (Amp *a*)	−1.793 ± 0.167	−1.860 ± 0.184	−1.912 ± 0.202	−1.884 ± 0.169	0.097	$0.006^{*,†}$	$0.017^{*}$	0.040
$f_{6,\text{PPG}}$ (Ratio b/a)	0.718 ± 0.176	0.669 ± 0.163	0.602 ± 0.196	0.647 ± 0.168	0.173	$0.008^{*,†}$	0.077	0.086
$f_{7,\text{PPG}}$ (Slope ab)	0.014 ± 0.010	0.019 ± 0.012	0.023 ± 0.014	0.021 ± 0.012	0.056	$0.004^{**,†}$	$0.006^{*,†}$	0.022
$f_{1,\mathrm{APG}}$ (Energy ab)	3.6$\times 10^{5}$ ± 4.7$\times 10^{5}$	6.4$\times 10^{5}$ ± 6.6$\times 10^{5}$	1.1$\times 10^{6}$ ± 1.2$\times 10^{6}$	6.2$\times 10^{5}$ ± 5.4$\times 10^{5}$	$0.02^{*}$	8.9$\times 10^{‒4**,†}$	$0.024^{*}$	1.5$\times 10^{‒2}$
$f_{2,\mathrm{APG}}$ (Energy aa)	8.0$\times 10^{5}$ ± 1.1$\times 10^{6}$	1.6$\times 10^{6}$ ± 1.7$\times 10^{6}$	2.5$\times 10^{6}$ ± 2.7$\times 10^{6}$	1.7$\times 10^{6}$ ± 1.5$\times 10^{6}$	$0.01^{*}$	1.1$\times 10^{‒3**,†}$	3.4$\times 10^{‒3**,†}$	5.9$\times 10^{‒3}$
$f_{3,\mathrm{APG}}$ (Energy ba)	4.6$\times 10^{5}$ ± 6.2$\times 10^{5}$	9.7$\times 10^{5}$ ± 1.1$\times 10^{6}$	1.4$\times 10^{6}$ ± 1.6$\times 10^{6}$	1.2$\times 10^{6}$ ± 1.1$\times 10^{6}$	$0.006^{*}$	1.1$\times 10^{‒3**,†}$	1.5$\times 10^{‒3**,†}$	3.0$\times 10^{‒3}$
$f_{4,\mathrm{APG}}$ (Amp *b*)	−106.723 ± 78.852	−161.405 ± 100.373	−197.465 ± 128.413	−166.542 ± 94.505	$0.01^{*}$	7.2$\times 10^{‒4**,†}$	5.5$\times 10^{‒3**,†}$	5.4$\times 10^{‒3}$
$f_{5,\mathrm{APG}}$ (Amp *a*)	103.586 ± 74.426	154.279 ± 98.926	193.623 ± 125.186	164.371 ± 94.626	$0.01^{*}$	6.9$\times 10^{‒4**,†}$	3.5$\times 10^{‒3**,†}$	5.9$\times 10^{‒3}$
$f_{6,\mathrm{APG}}$ (Ratio b/a)	−1.015 ± 0.156	−1.057 ± 0.140	−1.023 ± 0.088	−1.026 ± 0.094	0.12	0.641	0.634	0.465
$f_{7,\mathrm{APG}}$ (Slope ab)	−5.763 ± 4.231	−9.469 ± 6.472	−11.165 ± 7.785	−10.370 ± 6.219	$0.0052^{*}$	5.8$\times 10^{‒4**,†}$	9.5$\times 10^{‒4**,†}$	2.2$\times 10^{‒3}$

**Table 3:** Leave-one-out heat stress classification performance on PPG-chemical, biological and radiological (CBR) responders in the tropical condition database. Four classification methods are used in this analysis: Mahalanobis, linear discriminant analysis (LDA), quadratic discriminant analysis (QDA) and the linear support vector machine (SVM). The PPG signals were collected from 40 heat-acclimatized emergency responders for 20 s during the 10-min break of each exercise (*cf.* Figure 2). To evaluate the performance of each feature, we applied the $F_{1}$ score, which is defined as 2×(SE×PP)/(SE+PP) (SE, sensitivity; PP, positive predictivity). Here, OA stands for overall accuracy (average of $F_{1}$ scores for all classifiers).

Feature	Before Exercise *vs.* After Exercise 1				Before Exercise *vs.* After Exercise 2				Before Exercise *vs.* After Exercise 3				OA
	Mahalanobis	LDA	QDA	SVM	Mahalanobis	LDA	QDA	SVM	Mahalanobis	LDA	QDA	SVM	(%)
$f_{1,\text{PPG}}$ (Energy ab)	50.00	55.00	40.96	59.79	60.24	60.98	61.33	62.16	64.86	63.01	61.97	63.01	58.61
$f_{2,\text{PPG}}$ (Energy aa)	72.73	72.73	72.73	75.00	83.54	78.95	78.95	82.93	82.93	82.67	84.62	85.00	79.40
$f_{3,\text{PPG}}$ (Energy ba)	71.79	71.79	71.79	78.05	82.05	80.52	80.52	84.34	80.49	76.32	77.92	79.01	77.88
$f_{4,\text{PPG}}$ (Amp *b*)	53.93	57.14	46.58	58.14	65.12	65.12	64.44	65.17	61.73	61.73	57.14	62.79	59.92
$f_{5,\text{PPG}}$ (Amp *a*)	61.36	61.36	63.04	63.04	67.44	67.44	63.74	67.44	67.47	67.47	65.88	65.85	65.13
$f_{6,\text{PPG}}$ (Ratio b/a)	59.52	60.24	49.35	59.52	65.85	65.85	67.42	65.85	64.20	64.20	60.76	62.50	62.11
$f_{7,\text{PPG}}$ (Slope ab)	62.07	63.64	62.50	62.50	66.67	68.18	67.39	66.67	69.05	69.05	68.97	69.05	66.31
$f_{1,\mathrm{APG}}$ (Energy ab)	61.05	61.05	69.31	69.31	67.42	71.58	70.59	69.57	65.91	65.91	65.22	68.04	67.08
$f_{2,\mathrm{APG}}$ (Energy aa)	63.16	62.50	70.59	69.90	66.67	72.34	70.59	72.34	68.18	67.42	72.92	68.82	68.78
$f_{3,\mathrm{APG}}$ (Energy ba)	63.83	63.92	70.59	70.59	68.13	72.92	69.90	73.47	70.45	71.11	74.23	71.74	70.07
$f_{4,\mathrm{APG}}$ (Amp *b*)	64.37	63.64	63.16	63.16	68.29	70.59	71.58	68.97	67.47	67.47	68.97	69.05	67.22
$f_{5,\mathrm{APG}}$ (Amp *a*)	62.92	64.44	62.50	63.16	72.29	68.97	70.83	68.18	67.44	67.44	68.18	68.97	67.11
$f_{6,\mathrm{APG}}$ (Ratio b/a)	54.12	54.76	53.73	50.70	67.24	53.01	35.09	54.35	69.09	53.66	32.79	51.28	52.49
$f_{7,\mathrm{APG}}$ (Slope ab)	65.91	65.93	69.31	68.00	73.17	70.33	73.47	71.11	69.77	71.26	75.27	72.73	70.52

After applying the leave-one-out cross-validation, the energy of aa scored the highest overall accuracy (OA=79.40%), while the energy of ba scored the second highest overall accuracy (OA=77.88%). Now, after using four different classifiers in all heat stress simulation stages, we have clear evidence that the energy of aa is the optimal feature for detecting heat stress.

**Figure 6:**
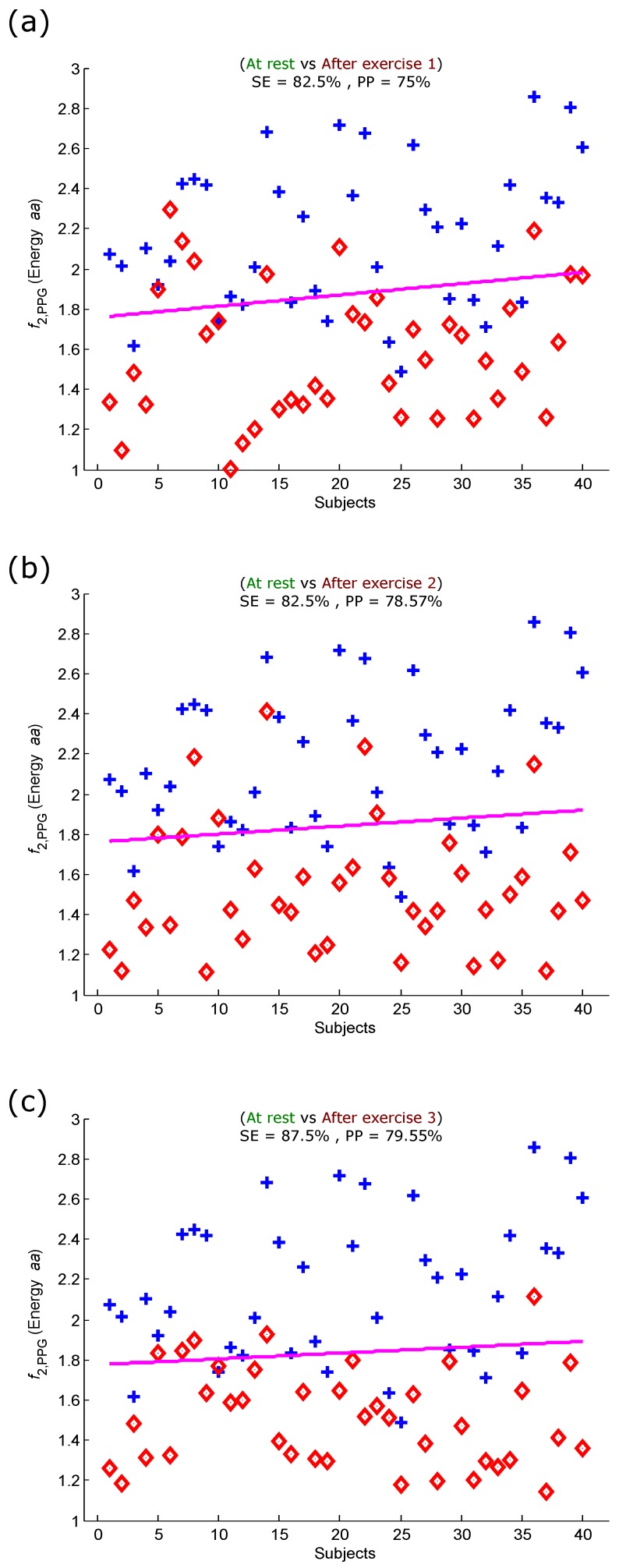
Linear classification of PPG signals measured before and after the simulated heat stress induction based on the energy of the aa area of the PPG signal. (**a**) At rest *vs.* after exercise 1; (**b**) at rest *vs.* after exercise 2; (**c**) at rest *vs.* after exercise 3. Here, SE stands for sensitivity and PP stands for positive predictivity. The plus signs refer to subjects measured at rest, while the diamond signs refer to subjects measured after the simulated heat stress induction.

Figure 6 shows the LDA classification performance of the optimal feature for detecting heat stress after three exercises. The optimal feature (energy of aa) detected heat stress after the first exercise with a SE of 82.5% and PP of 75%, as shown in Figure 6a. The PP was slightly improved in classifying after exercise 2 (*cf.* Figure 6b); however, the detection of heat stress was considerably improved after exercise 3 with an SE of 87.5% and a PP of 79.55%, as shown in Figure 6c. Interestingly, the overall accuracy of detecting heat stress increased as the exercise progressed from one simulated heat stress induction stage to the next. This new outcome demonstrates that the energy of the aa area can be used to assess heat stress without the need for measuring the heart rate, which can be used as an indicator of heat stress tolerance.

**Figure 7:**
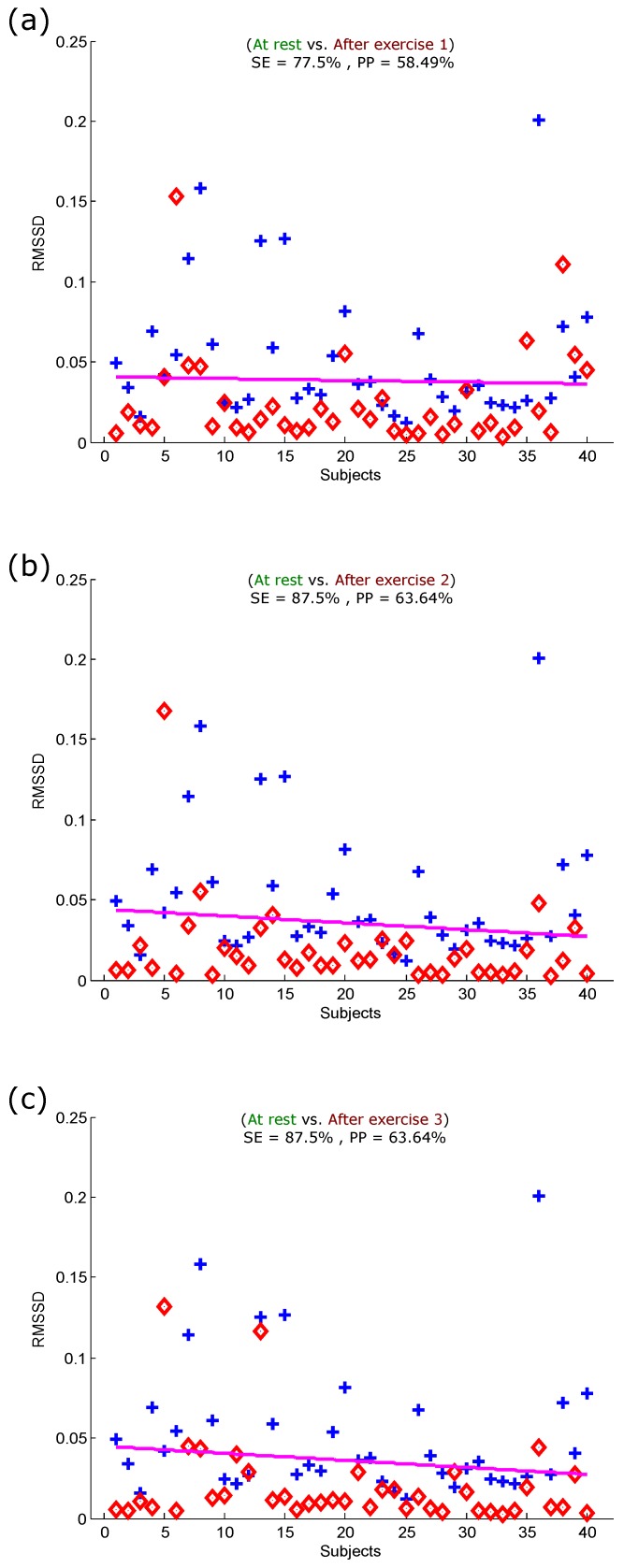
Linear classification of PPG signals measured before and after the simulated heat stress induction based on the RMSSD. (**a**) At rest *vs.* after exercise 1; (**b**) at rest *vs.* after exercise 2; (**c**) at rest *vs.* after exercise 3. Here, RMSSD stands for the square root of the mean of the squares of the successive aa (heartbeats) intervals; SE stands for sensitivity; and PP stands for positive predictivity. The plus signs refer to subjects measured at rest, while the diamond signs refer to subjects measured after the simulated heat stress induction.

**Figure 8:**
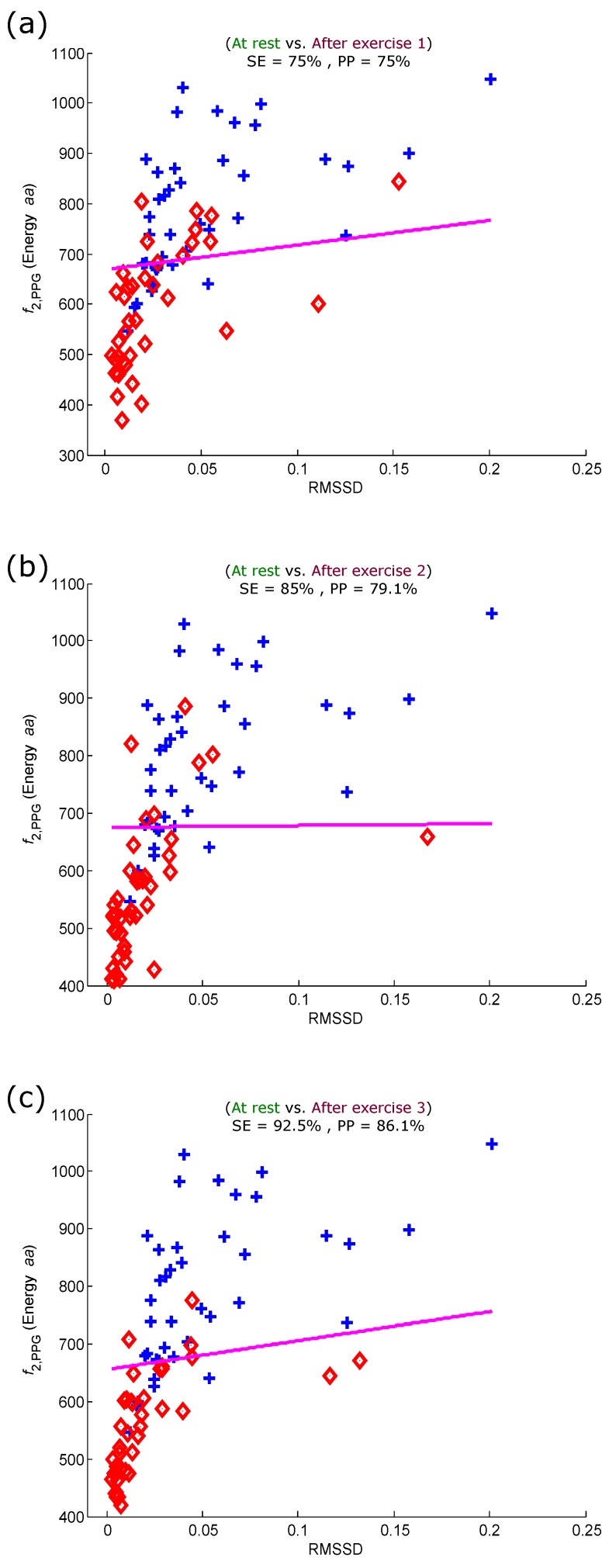
Linear classification of PPG signals measured before and after the simulated heat stress induction based on the energy of the aa area of the PPG signal and RMSSD. (**a**) At rest *vs.* after exercise 1; (**b**) at rest *vs.* after exercise 2; (**c**) at rest *vs.* after exercise 3. Here, RMSSD stands for square root of the mean of the squares of the successive aa (heartbeats) intervals; SE stands for sensitivity; and PP stands for positive predictivity. The plus signs refer to subjects measured at rest, while the diamond signs refer to subjects measured after the simulated heat stress induction.

To ensure that the energy of the aa area can be used to replace other measures of heat stress, it is important to compare its performance with another heat stress index, such as BCT and RMSSD. The problem is we could not measure BCT immediately after exercise, and a few subjects had to wait, which cooled them down a bit (*cf.* Figure 4). Therefore, we had to compare the optimal feature with the RMSSD calculated from the PPG signals. Figure 7 shows the performance of the RMSSD for detecting all simulated heat stress inductions. The RMSSD detected heat stress after the first exercise with an SE of 77.5% and a PP of 58.49%, as shown in Figure 7a. The detection of heat stress was considerably improved after exercise 2 and exercise 3 with an SE of 87.5% and a PP of 63.64%, as shown in Figure 7b,c. It is clear that the optimal feature (the energy of aa) outperformed the RMSSD heat stress index. Note that the RMSSD showed no classification improvement in detecting heat stress after exercise 2 and Exercise 3: the sensitivity and positive predictivity remained the same (*cf.* Figure 7b,c).

To maximize the heat stress detection performance, we combined the optimal feature (energy of aa) and the traditional HRV heat stress index (RMSSD). Figure 8 shows the performance of the combined features (energy of aa and RMSSD) for detecting all simulated heat stress inductions. The RMSSD detected heat stress after the first exercise with an SE of 75% and a PP of 75%, as shown in Figure 8a. The detection of heat stress was considerably improved after exercise 2, with an SE of 85% and a PP of 79.1%, as shown in Figure 8b. The progression of detecting heat stress continued, and the accuracy improved after exercise 3 with an SE of 92.5% and a PP of 86.1%, as shown in Figure 8c. It is clear that the use of combined features noticeably improved the heat stress detection performance with each sequential heat stress simulation induction stage.

## 4. Limitations of the Study and Future Work

Exploring these findings across different genders, ages and health condition groups will improve the generalization across the population. To our knowledge, there is no available PPG database measured in tropical conditions or after heat stress that would allow a more thorough assessment and comparison of the tested algorithms. In future studies, it may be advisable to have multiple PPG systems to collect the signal immediately after exercise. In the present study, data were collected immediately after exercise. However, some participants may have cooled down while queuing for measurement.

## 5. Conclusions

The findings of this preliminary study indicate that heat stress can be assessed using the blood volume of PPG signals. This new outcome shows that combining the energy of the aa area with the RMSSD of the PPG signals can be used to assess heat stress and can be used as an indicator of heat stress tolerance. The results of this study indicate that PPG can be a potential modality for heat stress analysis and identification of individuals at risk. Our preliminary study demonstrates indicative results and now motivates the need for a larger study that validates the energy of the aa area from the PPG against traditional heat stress tolerance indices. The developed classifier can then be embedded in wearable PPG sensors to ultimately detect the stress level in real time.
